# The value of diffusion kurtosis imaging in assessing pathological complete response to neoadjuvant chemoradiation therapy in rectal cancer: a comparison with conventional diffusion-weighted imaging

**DOI:** 10.18632/oncotarget.17491

**Published:** 2017-04-27

**Authors:** Feixiang Hu, Wei Tang, Yiqun Sun, Dang Wan, Sanjun Cai, Zhen Zhang, Robert Grimm, Xu Yan, Caixia Fu, Tong Tong, Weijun Peng

**Affiliations:** ^1^ Department of Radiology, Fudan University Shanghai Cancer Center, Department of Oncology, Shanghai Medical College, Fudan University, Shanghai, P.R. China; ^2^ Department of Colorectal Surgery, Fudan University Shanghai Cancer Center, Department of Oncology, Shanghai Medical College, Fudan University, Shanghai, P.R. China; ^3^ Department of Radiotherapy, Fudan University Shanghai Cancer Center, Department of Oncology, Shanghai Medical College, Fudan University, Shanghai, P.R. China; ^4^ MR Applications Predevelopment, Siemens Healthcare GmbH, Erlangen, Germany; ^5^ MR Collaboration NE Asia, Siemens Healthcare, Shanghai, P.R. China; ^6^ APPL, Siemens Shenzhen Magnetic Resonance Ltd., Shenzhen, P.R. China

**Keywords:** diffusion kurtosis imaging, apparent diffusion coefficient, neoadjuvant chemoradiation therapy, locally advanced rectal cancer, pathological complete response

## Abstract

**Objectives:**

The aim of this study is to comprehensively evaluate the advantage of diffusion kurtosis imaging (DKI) in distinguishing pathological complete response (pCR) from non-pCR patients with locally advanced rectal cancer (LARC) after neoadjuvant chemoradiation therapy (CRT) in comparison to conventional diffusion-weighted imaging (DWI).

**Material and Methods:**

Fifty-six consecutive patients diagnosed with LARC were prospectively enrolled and underwent pre- and post-CRT MRI on a 3.0 T MRI scanner. Apparent diffusion coefficient (ADC), mean diffusion (MD) and mean kurtosis (MK) values of the tumor were measured in pre- and post-CRT phases and then compared to histopathologic findings after total mesorectal excision (TME). Both Mann-Whitney U-test and Kruskal-Wallis test were used as statistical methods. Diagnostic performance was determined using receiver operating characteristic (ROC) curve analysis.

**Results:**

For a total of 56 rectal lesions (pCR, *n* = 14; non-pCR, *n* = 42), the MK_pre_ and MK_post_ values were much lower for the pCR patients (mean±SD, 0.72±0.09 and 0.56±0.06, respectively) than those for the non-pCR patients (0.89±0.11 and 0.68±0.08, respectively) (*p* < 0.001). The ADC_post_ and the change ratio of apparent diffusion coefficient (ADC_ratio_) values was significantly higher for the pCR patients (mean±SD, 1.31±0.13 and 0.64±0.34, respectively) than for the non-pCR patients (1.12±0.16 and 0.33±0.27, respectively) (*p* < 0.001 and *p* = 0.001, respectively). In addition, the MD_post_ and the change ratio of mean diffusion (MD_ratio_) (2.45±0.33 *vs.* 1.95±0.30, *p* < 0.001; 0.80±0.43 *vs.* 0.35±0.32, *p* < 0.001, respectively) also increased, whereas the ADC_pre_, MD_pre_ and the change ratio of mean kurtosis (MK_ratio_) of the pCR (0.82±0.11, 1.40±0.21, and 0.23±0.010, respectively) exhibited a neglectable difference with that of the non-pCR (*p* = 0.332, 0.269, and 0.678, respectively). The MK_post_ showed relatively high sensitivity (92.9%) and high specificity (83.3%) in comparison to other image indices. The area under the receiver operating characteristic curve (AUROC) that is available for the assessment of pCR using MK_post_ (0.908, cutoff value = 0.6196) were larger than other parameters and the overall accuracy of MK_post_ (85.7%) was the highest.

**Conclusions:**

Both DKI and conventional DWI hold great potential in predicting treatment response to neoadjuvant chemoradiation therapy in rectal cancer. The DKI parameters, especially MK_post_, showed a higher specificity than conventional DWI in assessing pCR and non-pCR in patients with LARC, but the pre-CRT ADC and MD are unreliable.

## INTRODUCTION

Magnetic resonance imaging (MRI) has been widely used to evaluate the neoadjuvant CRT response in rectal cancer because of its classical morphological MR evaluation [1-3]. Quantitative biomarkers of functional magnetic resonance imaging could objectively reflect the response to therapy [4-8] and play an essential role in identifying patients with good prognosis. For instance, patients who might benefit from surgery alone could merely avoid long-term exposure to the toxicity of radiotherapy (RT) [9, 10], whereas those with pCR could benefit from either less invasive surgery (*i.e.*, transanal endoscopic microsurgery, TEM) [11, 12] or a “wait-and-see” strategy [13, 14]. In addition, patients with no response to the treatment (non-responders [NRs]) that were identified at an early stage (2-3 weeks after the start of neoadjuvant CRT) might have chance to choose alternative treatment [15, 16]. However, the current lack of reliable non-invasive diagnostic tools to identify complete responders remains a major challenge [17].

A consensus on the ADC threshold in distinguishing pCR from non-pCR remains controversial. Kim SH *et al.* found that post-CRT ADC values could reliably differentiate pCR from non-pCR in LARC [18], whereas Curvo-Semedo L *et al.* noted that ADC measurements were not accurate for assessing a CR [19]. The conventional DWI model is based on the assumption that water diffusion within a voxel has a single component and follows a Gaussian behavior that water molecules diffuse without any restriction [20]. However, due to the presence of microstructures (i.e., two tissue types or components within one voxel, and organelles and cell membranes), random motion or diffusion of thermally agitated water molecules within biologic tissues exhibits a non-Gaussian phenomena [21]. A non-Gaussian diffusion model called as diffusion kurtosis imaging was proposed by Jensen and his co-workers in 2005 [22]. This model calculates the kurtosis coefficient (K) that signifies the deviation of tissue diffusion from a Gaussian model, and the diffusion coefficient(D) with the correction of non-Gaussian bias.

Several studies reported that DKI performed better than conventional ADC in tumor detecting and grading [23-29]. It is reported that DKI was more applicable and appropriate for assessing early response to neoadjuvant chemotherapy (NAC) in patients with locally advanced nasopharyngeal carcinoma (NPC) than ADC [30].The results showed that ∆D (day4) was more sensitive in predicting the treatment results (*P* = 0.006). Recently, one study reported the application of DKI in rectal cancer before and after CRT [31]. This study evaluated the feasibility of DKI in assessing treatment response (patients with pTRG-1 or pTRG-2 were classified as good responders, whereas the remaining patients with pTRG-3-5 scores were classified as poor responders) to neoadjuvant chemoradiotherapy (CRT) in patients with LARC. Thus, the aim of our study is to determine whether DKI can perform better in predicting and evaluating pCR in patients with LARC after neoadjuvant CRT than conventional DWI.

## MATERIALS AND METHODS

### Patients

Between January 2014 and September 2015, 60 consecutive patients were prospectively enrolled, and the patients were histologically confirmed primary rectal adenocarcinoma and locally advanced disease, which includes T3 and T4 stages on MR images, and/or N-category positive. The exclusion criteria followed the several points: (a) MRI contraindications (e.g., aneurysm clip, metal prosthesis) (*n* = 0); (b) incomplete MRI and pathological data (*n* = 1); (c) delayed (time between second MRI and surgery was more than 1 month) or cancelled surgery (*n* = 2); (d) hypersensitivity to the study drug or to one of the excipients (*n* = 0). Besides, patients were excluded if they were treated with prior hormonal and/or radiation or they participated in another clinical trial (*n* = 1). Thus, 56 patients (mean age ± standard deviation: 52.1 years± 11.4; range, 25-70 years) with LARC after CRT were enrolled in the final study population.

### Study protocol

All patients underwent pre-CRT MR imaging (2-5 days before CRT) for primary tumor staging and a second restaging MR imaging examination (1-4 days before surgery) for response evaluation. For patients with neoadjuvant chemotherapy, the interval between neoadjuvant therapy and surgery was 6 ∼ 8 weeks. Afterwards, patients underwent standardized surgical excision using the TME technique, and the gross specimen evaluation was carried out by one gastrointestinal histopathologist.

### MR examination

MRI examinations were performed on a 3.0 T MRI scanner (MAGNETOM Skyra, Siemens Healthcare, Erlangen, Germany) with a 16-channel phase-array body coil. The MRI protocol included a sagittal T2-weighted TSE (turbo spin echo), an oblique axial thin-section T2-weighted TSE and an oblique axial multi-bEPI (single-shot echo-planar-imaging) DWI sequence. The detailed parameters of the DWI sequence were as follows: TR/TE = 4500/82 ms; FOV = 200x180 mm^2^; slice thickness =6 mm; scan matrix = 140x140; voxel size = 1.4x1.4x6 mm^3^,phase oversampling = 20%; no. of slices = 20; tri-directional diffusion gradients were performed with b values of 0, 700, 1400, and 2100 s/mm^2^ (with NEX = 1, 2, 4, 6, respectively); GRAPPA acceleration factor = 2; and acquisition time =3 min 51 sec. Patients did not receive bowel preparation (no endorectal filling, an enema or using spasmolytics) before the MR examinations.

### Neoadjuvant CRT

Radiation therapy was performed using a three-dimensional conformational multiple field technique. A dose of 45 Gy (1.8 Gy per day, 5 days per week, for 5 weeks) was delivered to the entire pelvis. In addition, a dose from 5.4 to 9 Gy (3-5 days, 1.8 Gy/day) was imposed on the tumor volume with 6 to 15 MV energy photons. Chemotherapy was delivered concomitantly to radiation therapy, which was consisting of two-hour oxaliplat in infusion (50 mg/m^2^) on the first day of each week during radiotherapy and five daily continuous infusions of 5-fluorouracile (200 mg/m^2^/d). The second MRI was performed to assess the response in patients after completion of neoadjuvant CRT (averaging 7 weeks). The surgery was performed within 4 days after second MRI.

### Surgical technique

TME was used in all patients and followed a standardized technique [32]. The operation was implemented by a skillful colorectal surgeon with over 25 years practice in the TME technique.

### Histopathological evaluation

The basic histopathology evaluation of the primary tumor (including type and grade of the tumor) after post-surgery resection treatment was assessed by one experienced gastrointestinal histopathologist. A correlation between imaging and pathology in the entirely irradiated area was also evaluated by observing the intestinal segment containing the neoplasm that could be obtained by sectioning orthogonal to the long axis and acquiring macro-section specimens of 2 - 3 mm thickness. All TNM statuses were obtained basing on the American Joint Committee on Cancer (AJCC, the latest 7th edition) staging system [33]. Tumor regression was graded as follows [34]: Patients with pTRG 0 or pTRG 1 were classified as good responders (no remaining viable cancer cells; only small clusters or single cancer cells), whereas the remaining patients with pTRG 2-3 were classified as poor responders (presence of residual cancer with dominant fibrosis; minimal or no tumor death, extensive residual cancer). Regression grading involved the primary tumor and regional lymph nodes. Downstaging was determined by comparing the pretreatment and postoperative pathologic classifications and defined as ypStage 0-I (ypT0-2N0M0; the “yp” prefix indicates final staging after CRT [y] and postoperative pathologic examination [p]). If no tumor cells were identified in the resected specimen and only fibrotic mass or acellular mucin pools were present, the type of response was considered as complete response (ypT0N0) and the patient was labeled as pCR.

### Image analysis

The parameter maps of both DKI and conventional DWI were obtained from the multi-b DWI data with all measured b values using the prototype post-processing software Body Diffusion Toolbox (Siemens Healthcare GmbH, Erlangen, Germany). Diffusion kurtosis imaging was imported into the software in order to obtain the final fitted images (ADC map, MD map, and MK map). ROIs (regions of interest) were manually drawn on the each cross-sectional area of the primary lesions by two radiologists in consensus, simultaneously avoiding to encircle distortion artifacts and macroscopically visible necrotic or cystic portions areas in the axial ADC map deriving from T2-weighted images. One professor with over 10 years of clinical experience, and a less experienced professor with 5 years of clinical experience in interpreting rectal MR imaging studies were blinded to analysis the histopathological results. Then, ROIs were automatically circled on D map and K map by the software. After the completion of therapy, supposing there was no visible residual tumor, particularly in those patients with a pCR after neoadjuvant CRT, the ROIs were depicted in the same area that was considered to be the normal residual rectum, concurrently using pre-treatment ROIs as a reference. The multi-b DW images were obtained by fitting of voxel-by-voxel using the DK signal decay equation by a two-variable linear least squares algorithm as used in previous study [21]:

$$S\left(b\right)=S_{0}\times \exp\left(-\mathrm{bD}+16b^{2}D^{2}K\right)$$ (1)

In this equation, S(b) is the signal intensity at a certain b-value; S_0_ is the baseline signal without diffusion weighting; D is a corrected diffusion coefficient; and K is the excess diffusion kurtosis coefficient. K describes the degree that molecular motion deviates from the perfect Gaussian distribution. When K is equal to 0, equation (1) is evolved into a conventional monoexponential equation:

$$S\left(b\right)=S_{0}\times \exp\left(-b\times \mathrm{ADC}\right).$$ (2)

The difference between D and ADC is that D is a corrected form of ADC for use in non-Gaussian circumstances.

The change ratios of MK, MD and ADC before and after CRT were calculated according to the following equations:

$$\begin{array}{l} {\mathrm{ADC}}_{\text{ratio}}=({\mathrm{ADC}}_{\text{post}}-{\mathrm{ADC}}_{\text{pre}})/{\mathrm{ADC}}_{\text{pre}};\\ {\mathrm{MD}}_{\text{ratio}}=\left({\mathrm{MD}}_{\text{post}}-{\mathrm{MD}}_{\text{pre}}\right)/{\mathrm{MD}}_{\text{pre}};\\ {\mathrm{MK}}_{\text{ratio}}=\left({\mathrm{MK}}_{\text{pre}}-{\mathrm{MK}}_{\text{post}}\right)/{\mathrm{MK}}_{\text{post}}. \end{array}$$

Where MK_pre_, MK_post_, MD_pre_, MD_post_, ADC_pre_, and ADC_post_ refer to MK, MD and ADC values before and after CRT,respectively.

### Statistical analysis

Two softwares, *i.e.*, SPSS Statistics 21.0 (IBM Corp., Armonk, NY, USA) and Medcalc 12.7.2 (Medcalc software, Ostend, Belgium), were used for statistical data analysis. Continuous variables were presented as the mean ± standard deviation (SD). The DWI and DKI parameters of patients with pCR (*n* = 14) and non-pCR (*n* = 42) were compared with each other using the nonparametric Mann-Whitney U test. The Kruskal-Wallis test was used to assess differences between the following paired groups: pCR *vs*. non-pCR, good regression (TRG0-1) *vs*. poor regression (TRG2-3) and downstaging *vs*. non-downstaging. As well, ROC curves were depicted to characterize each parameter value for evaluating the CRT outcome. The optimal cut-off values (obtained according to the maximal Youden index = sensitivity + specificity-1), the corresponding sensitivity, specificity, positive predictive value (PPV), negative predictive value (NPV) and accuracy could be calculated.

## RESULTS

### Patient characteristics

**Figure 1:**
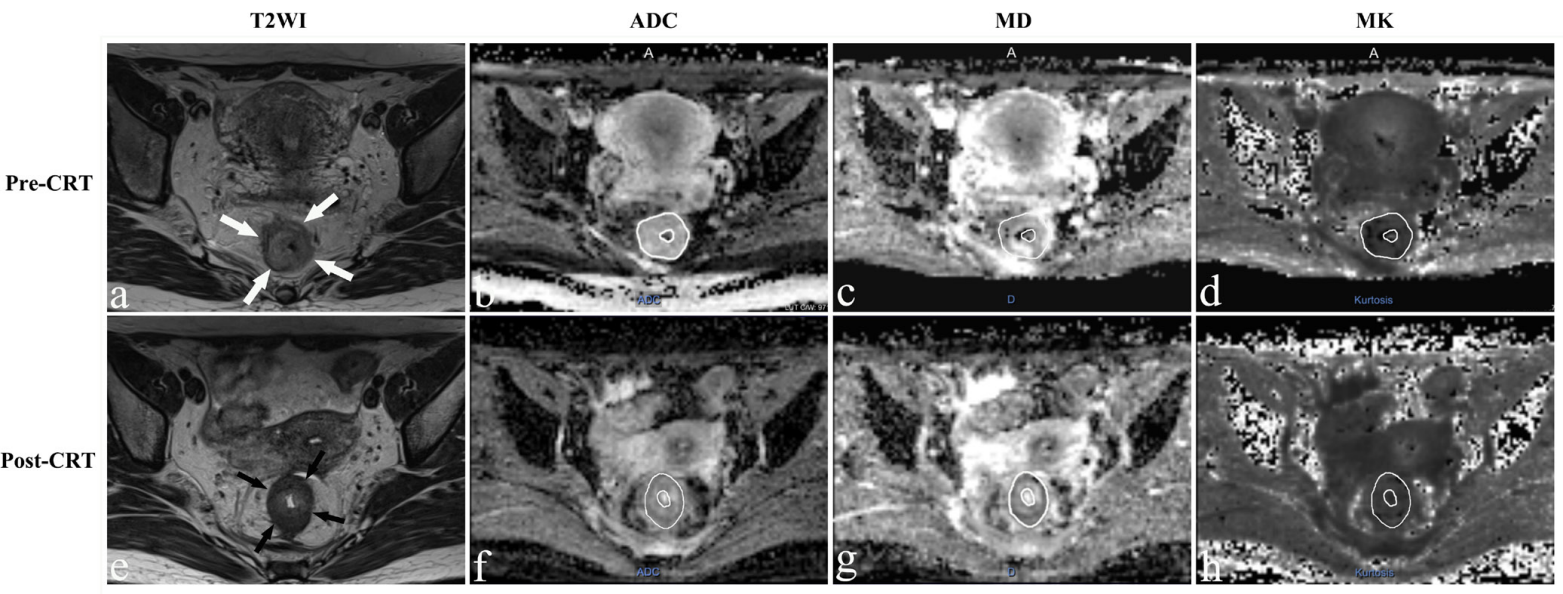
Representative images of a 36-year-old woman with a pCR Images in each row are from two measurement time points: 2-5 days before CRT (pre-CRT) and 1-4 days before surgery (post-CRT). **a.**, **e.** Before and after CRT T2-weighted MR image shows that there is a tumor with ring shape visible in the rectum (arrows). The ADC map, D map, and K map have similar image contrast and manual tracing of ROIs within the tumor area (The whole tumor was not shown here). The ADC, MD and MK values were 0.932×10^-3^ mm^2^/s, 1.580×10^-3^ mm^2^/s and 0.657 before treatment. The ADC and MD values increased obviously to 1.240×10^-3^mm^2^/s and 2.601×10^-3^mm^2^/s, while the MK value decreased slightly to 0.535 after CRT.

**Figure 2:**
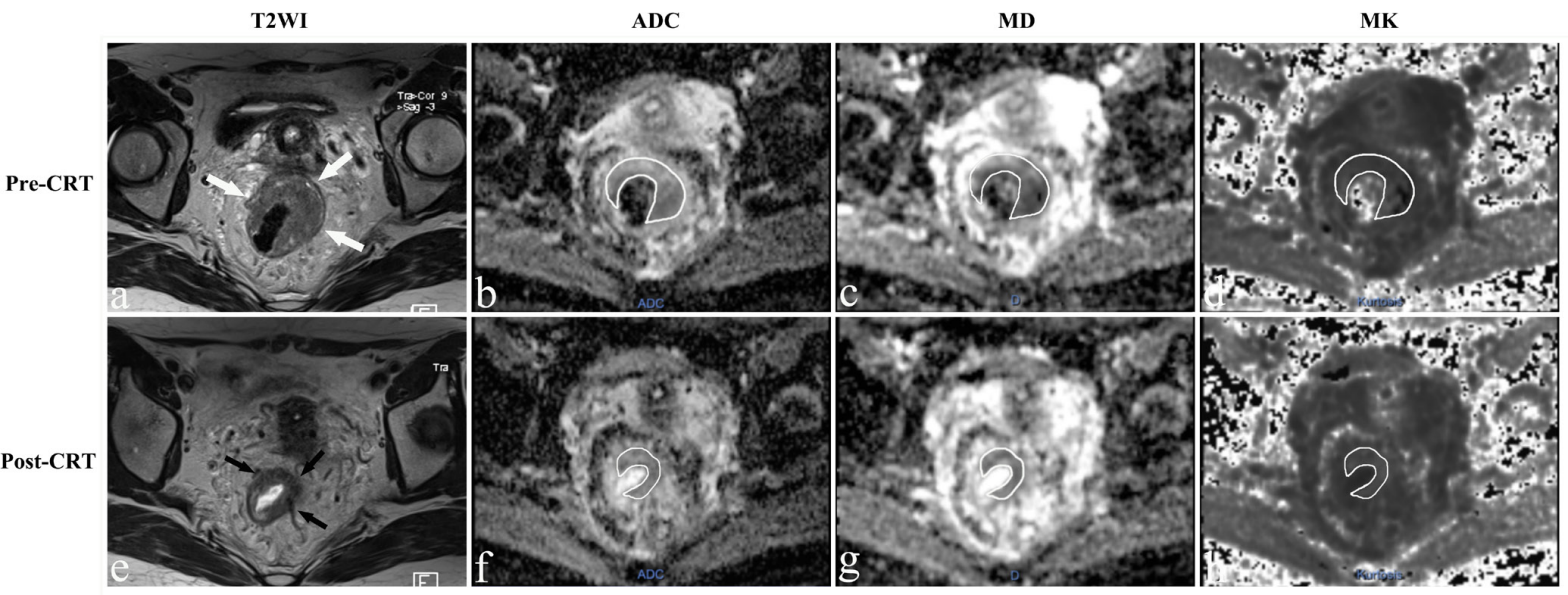
MR images of a 43-year-old woman with a non-pCR **a.**-**e.** T2-weighted MR image before and after CRT shows that there is a visible tumor with a horseshoe shape in the rectum (arrows). The ADC, MD and MK values were 0.924×10^-3^ mm^2^/s, 1.424×10^-3^ mm^2^/s, and 0.876 before treatment. After CRT, the ADC and MD increased slightly to 0.947×10^-3^mm^2^/s and 1.533×10^-3^mm^2^/s, respectively, and the MK value decreased slightly to 0.812.

The population was consisting of 56 patients (16 females, 40 males) with an average age of 52.1±11.4 years. Fourteen patients showed pCR (Figure 1), whereas 42 patients were classified as non-pCR (Figure 2).

### Parameters between pCR and non-pCR

The MK_pre_ and MK_post_ values in patients with pCR were much lower than that in non-pCR, respectively, *e.g.*, 0.72±0.09 *vs.* 0.89±0.11, *p* < 0.001 and 0.56±0.06 *vs.* 0.68±0.08, *p<*0.001, whereas no significant difference in the MK_ratio_ between the pCR and non-pCR was observed (0.21±0.13 and 0.23±0.01, *p* = 0.678). The ADC_pre_ and MD_pre_ values between pCR and non-pCR patients also exhibited no significant difference(0.82±0.11 and 0.86±0.15, *p* = 0.332; 1.40±0.21 and 1.50±0.33, *p* = 0.269, respectively). Significant differences of either ADC_post_ or MD_post_ between pCR and non-pCR were found, and either ADC_ratio_ or MD_ratio_ exhibited a significant correlation in differentiating between pCR and non-pCR (*p<*0.001). Additional analysis (TRG and downstaging) was performed for all patients before and after CRT to complement the results (Table 1).

**Table 1:** Correlation between parameters and the different pathologic scoring systems

	pCR*		TRG0-1*		TNM-downstaging*	
Pathologic evaluation	Non-pCR(*n*=42) *vs*. pCR(*n*=14)	*p*	TRG2-3(*n*=34) *vs*.TRG0-1(*n*=22)	*p*	No(*n*=30) *vs*. Yes(*n*=26)	*p*
ADC_pre_(×10^-3^mm^2^/s)	0.86±0.15 vs. 0.82±0.11	0.332	0.86±0.17 vs. 0.83±0.11	0.524	0.85±0.13 vs. 0.85±0.17	0.944
ADC_post_(×10^-3^mm^2^/s)	1.12±0.16 vs. 1.31±0.13	<0.001	1.10±0.15 vs. 1.27±0.17	<0.001	1.10±0.15 vs. 1.25±0.17	0.001
ADC_ratio_	0.33±0.27 vs. 0.64±0.34	<0.001	0.32±0.26 vs. 0.55±0.34	0.006	0.32±0.24 vs. 0.52±0.35	0.015
MD_pre_(×10^-3^mm^2^/s)	1.50±0.33 vs. 1.40±0.21	0.269	1.51±0.35 vs. 1.43±0.21	0.314	1.49±0.27 vs. 1.46±0.34	0.763
MD_post_(×10^-3^mm^2^/s)	1.95±0.30 vs. 2.45±0.33	<0.001	1.98±0.32vs. 2.23±0.42	0.012	1.94±0.29 vs. 2.23±0.41	0.003
MD_ratio_	0.35±0.32 vs. 0.80±0.43	<0.001	0.36±0.32 vs. 0.62±0.46	0.017	0.35±0.31 vs. 0.59±0.45	0.017
MK_pre_	0.89±0.11 vs. 0.72±0.09	<0.001	0.90±0.11 vs. 0.77±0.11	<0.001	0.86±0.10 vs. 0.82±0.14	0.075
MK_post_	0.68±0.08 vs. 0.56±0.06	<0.001	0.69±0.07 vs. 0.59±0.08	<0.001	0.67±0.08 vs. 0.63±0.10	0.167
MK_ratio_	0.23±0.01 vs. 0.21±0.13	0.678	0.22±0.11 vs. 0.23±0.11	0.733	0.23±0.10 vs. 0.21±0.12	0.513

*Data are the means±standard deviation.

MK_ratio_=(MK_pre_-MK_post_)/MK_pre_; MD_ratio_=( MD_post_-MD_pre_)/MD_pre_;

ADC_ratio_=(ADC_post_-ADC_pre_)/ADC_pre_

### Diagnostic performance for assessment of pCR

**Figure 3:**
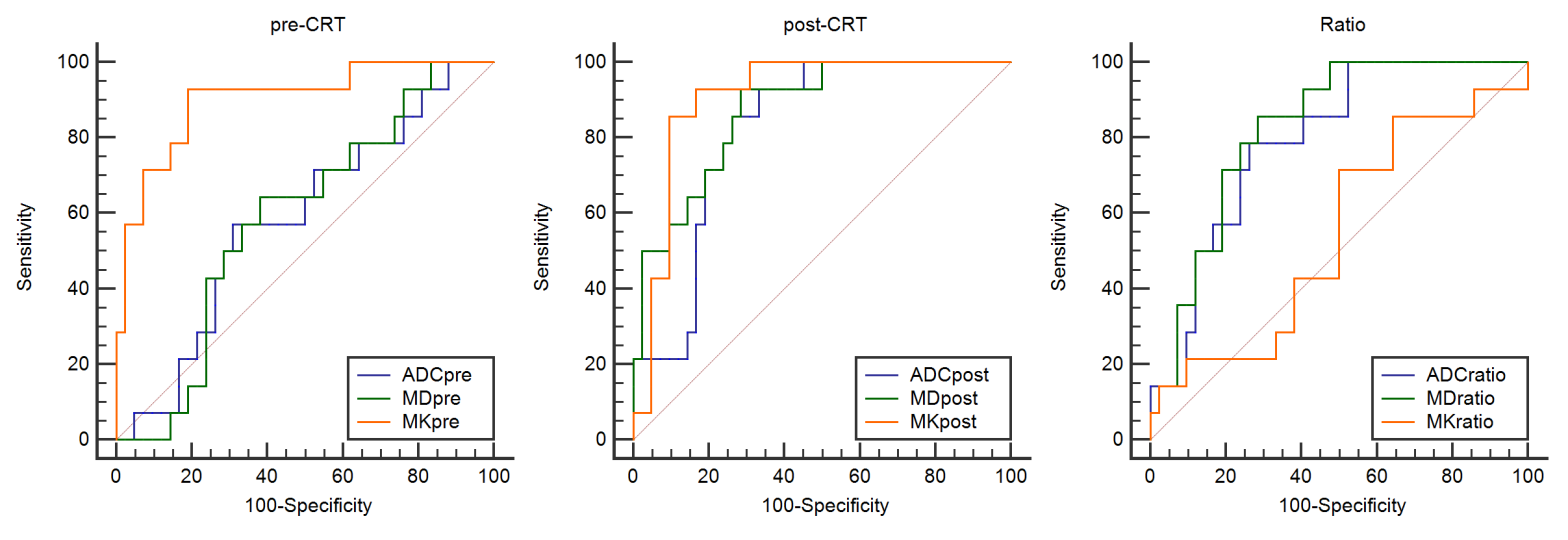
The receiver operating characteristic (ROC) curve analysis was performed to characterize each parameter for predicting the CRT outcome

The ROC curves were used to evaluate the diagnostic performance of DKI and conventional DWI in assessing a pCR (Fig. 3). ADC, MD, and MK located at the area under the curve were 0.583, 0.587, and 0.901 before CRT and 0.823, 0871, and 0.908 after CRT, respectively. Finally, the change ratio of ADC, MD, and MK showed that the ROC values were 0.793, 0.825, and 0.546, respectively. The optimal cutoff value for the accurate identification of patients with pathological CR was 0.6196 for MK_post_ (92.9% sensitivity, 83.3% specificity, 65% PPV, 97.2% NPV, and 85.7% accuracy, respectively) and 0.901 for MK_pre_ (92.9% sensitivity, 81% specificity, 61.9% PPV, 97.1% NPV, and 83.9% accuracy, respectively) (Table 2).

**Table 2:** Diagnostic performance for mean kurtosis and diffusion coefficients from DKI and ADC from DWI in detecting pCR

Factor	Sensitivity (%)	Specificity (%)	PPV (%)	NPV (%)	Accuracy (%)	AUC*	Optimal Cutoff^†^
Pre-CRT							
ADC	57.1 (8/14)	69 (29/42)	38.1 (8/21)	82.9 (29/35)	66.1 (37/56)	0.583 [0.44-0.71]	0.8178
MD	64.3 (9/14)	61.9 (26/42)	36 (9/25)	83.9 (26/31)	62.5 (35/56)	0.587 [0.45-0.72]	1.4505
MK	92.9 (13/14)	81 (34/42)	61.9 (13/21)	97.1 (34/35)	83.9 (47/56)	0.901 [0.79-0.97]	0.8199
Post-CRT							
ADC	92.9 (13/14)	66.7 (28/42)	48.1 (13/27)	96.6 (28/29)	73.2 (41/56)	0.823 [0.70-0.91]	1.1665
MD	92.9 (13/14)	71.43 (30/42)	52 (13/25)	96.8 (30/31)	76.8 (43/56)	0.871 [0.75-0.95]	2.0326
MK	92.9 (13/14)	83.3 (35/42)	65 (13/20)	97.2 (35/36)	85.7 (48/56)	0.908 [0.80-0.97]	0.6196
Ratio							
ADC	78.6 (11/14)	73.81 (31/42)	50 (11/22)	91.2 (31/34)	75 (42/56)	0.793 [0.66-0.89]	0.4321
MD	85.7 (12/14)	71.4 (30/42)	45.5 (10/22)	88.2 (30/34)	71.4 (40/56)	0.825 [0.70-0.91]	0.3439
MK	71.4 (10/14)	50 (21/42)	32.3 (10/31)	84 (21/25)	55.4 (31/56)	0.546 [0.41-0.68]	0.2343

Note.—Data are percentages, with numerators and denominators in parentheses.

^*^Data in square brackets are 95% CIs.

^†^Cutoff values were obtained by calculating the maximal Youden index: Youden index = sensitivity - (1- specificity).

PPV=Positive Predictive Value, NPV=Negative Predictive Value, AUC=Area Under the ROC Curve

## DISCUSSION

Clinicians may benefit from early prediction of the treatment response to LARC deliver individual treatment and avoid unnecessary systemic toxicity . Goshima *et al.* suggested that DKI is a new option for the assessment of post-therapeutic response of HCC [35]. Chen *et al.* indicated that DKI might perform better than monoexponential DWI in assessing early response to neoadjuvant chemotherapy for patients with locally advanced nasopharyngeal carcinoma [30]. Recently, Yu *et al.* suggested that DKI with entire-tumor histogram analysis was feasible and reliable for assessing the treatment response to neoadjuvant CRT and could be regarded as a promising tool for monitoring response to neoadjuvant CRT for patients with LARC [31]. They concluded that the change ratio of apparent diffusion applicable for Gaussian distribution (rΔDapp) deriving from the DK model provided substantial advantage for greater AUC and sensitivity for assessing treatment response to neoadjuvant CRT in comparison to mrTRG scores. In our study, both DKI and conventional DWI held the potential to predict the response to neoadjuvant chemoradiation therapy in rectal cancer. The DKI parameters, especially MK_post_, showed a higher specificity than conventional DWI for assessing pCR and non-pCR in patients with LARC. So far, there has been very little work, if any, to assess whether DKI in rectal cancer can be potentially used as an imaging biomarker of response to neoadjuvant CRT. Therefore, the aim of this investigation was to compare diffusion kurtosis imaging with conventional diffusion-weighted imaging for assessing the pathological complete response to neoadjuvant chemoradiation therapy in locally advanced rectal cancer.

The diffusion of water through a biologic tissue can be regarded as a random process and can be quantified by measuring the quantitative ADC. Several early studies suggested that the low ADC value in tissue was mainly attributed to the decreased interstitial space and the increased cellular density [36-38]. The ADC_post_ apparently increased in comparison to ADC_pre_ in both pCR and non-pCR patients. In our study, the ADC value increased from 0.85±0.16×10^-3^mm^2^/s in pre-CRT MR images to 1.17±0.18×10^-3^mm^2^/s in post-CRT MR images (*p* < 0.001). Effectively cytotoxic chemotherapy decreased tumor cellularity, which may lead to the increased diffusion in extracellular space, as reflected by the increased ADC values. A recent South Korea study found that the post-CRT ADC value reliably differentiated pCR from non-pCR in LARC [8]. Lambrecht *et al.* analyzed diffusion data of 20 patients with rectal cancer before and after CRT and acquired very high sensitivity (100%) and specificity (93%-100%) for pCR status when analyzing changes of ADC values before and after therapy [39]. In addition, they found that low pretreatment ADC values were significantly associated with pCR. Nevertheless, we failed to demonstrate advantage of ADC_pre_ measurements in differentiating between patients with pCR and non-pCR. Our study was consistent with another study wherein it is reported that the pre-CRT ADC of the pCR (0.85±0.10) showed no significant difference from that of the non-pCR (0.88±0.14) in LARC (*P* = 0.4094) [18]. The difference may result from the different drawing manners of the ROI, different combinations of b-values, tumor heterogeneity and different grouping methods. The MD values in our study are higher than the mean ADC values, which is consistent with most recent studies on DKI [26, 39, 40]. This increase can be attributed that conventional ADC is typically a sum of extra- and intracellular diffusion, whereas MD is mainly responsible for the extra-cellular portion [41]. Filli *et al.* compared the data sets of whole-body DKI and DWI, and suggested that whole-body DKI may more significantly reflect tissue’s microstructure than whole-body DWI did [42].

The K parameter represented the excessive diffusion kurtosis in the tissue and may be associated with microstructural complexity *in vivo* [22]. In our study, MK values before and after CRT in pCR were significantly lower than those in non-pCR patients. Tumor cells in non-pCR patients exhibited a higher cellularity with nuclear atypia. Conversely, tumor cells in some pCR patients with necrotic LARCs losed cellularity and usually generated liquefactive necrosis and local fibrosis, resulting in few diffusion barriers and increased structural complexity. Thus, employing the differences in MK values observed in our study to reflect the differences in microstructural complexity between pCR and non-pCR patients is possible.

The higher specificity in DKI model than that in the DWI model can be attributed to the several following aspects: (a) The conventional DWI model is based on the assumption that water diffusion within a voxel has a single component and follows a Gaussian behavior, whereas the DKI model is an attempt to account for the alteration of a normative pattern of distribution, provide a more accurate model of diffusion and capture the non-Gaussian diffusion behavior as a reflective marker for tissue heterogeneity [43]. (b) The cellular microstructure in lesions of non-pCR is more complex and more heterogeneous than that in pCR. (c) The pCR with liquefaction necrosis and fibrosis can reduce the local overlapping degree and cell density in each voxel, thus influencing water diffusion.

This preliminary pilot study encountered the following limitations. Firstly, the sample size was small, and the number of patients with pCR was low. Therefore, the larger sample sizes are needed to confirm our findings. Secondly, although MK exhibited feasible results for the assessment of complete remission of rectal cancer, there was still an overlap in MK values between the pCR and non-pCR. Thirdly, we only assessed the pCR patients experiencing pre- and post-CRT treatments, but failed to incorporate more time points into the study. Different periods of treatment should also be assessed. Finally, the reproducibility was also essential, according to a recent study [31]. We did not test the reproducibility, and the authors made an attempt to evaluate the feasibility of DKI in assessing the response from patients with LARC after neoadjuvant CRT and confirmed that DKI could be analyzed in a reproducible manner by different readers.

In conclusion, the calculated kurtosis value using the DKI model is demonstrated of a significantly higher specificity in differentiating pCR from non-pCR than that using ADCs. The MK_post_ can be regarded as the best diagnosis parameter to predict and evaluate pCR in LARC patients receiving preoperative CRT. If our results were substantiated in multicenter studies, DKI would be a new option for evaluating CRT response of LARC.
